# Immunogenicity when utilizing adenovirus serotype 4 and 5 vaccines expressing circumsporozoite protein in naïve and Adenovirus (Ad5) immune mice

**DOI:** 10.1186/1475-2875-11-209

**Published:** 2012-06-21

**Authors:** Nathaniel J Schuldt, Yasser A Aldhamen, Sarah Godbehere-Roosa, Sergey S Seregin, Youssef A Kousa, Andrea Amalfitano

**Affiliations:** 1 Genetics Program, Michigan State University, 2240 E Biomedical and Physical Sciences Building, East Lansing, MI, 48824, USA; 2 Department of Pediatrics, Michigan State University, East Fee Hall, East Lansing, MI, 48824, USA; 3 Department of Microbiology and Molecular Genetics, Michigan State University, 2215, USA Biomedical and Physical Sciences Building, East Lansing, MI, 48824, USA; 4 College of Osteopathic Medicine, Michigan State University, East Fee Hall, East Lansing, MI, 48824, USA

**Keywords:** Serotype 5, Serotype 4, Adenovirus, Malaria, Circumsporozoite protein, Vaccine, Heterologous, Homologous, Prime, Boost

## Abstract

**Background:**

Induction of potent long lasting effector T cell responses against liver stage malaria antigens strongly correlates with protection from malaria. While Adenovirus serotype 5 (Ad5) based malaria vaccine platforms have the ability to induce potent effector T cell responses against transgenes, high rates of pre-existing Ad5 immunity in malaria endemic regions has prompted study of alternative Ad serotype based malaria vaccines as replacements for Ad5 based malaria vaccines. The research described in this article examines the utility of alternative serotype adenovirus serotype 4 (Ad4) expressing a sporozoite surface protein (circumsporozoite protein (CSP)) (Ad4-CSP) to induce immune responses against CSP. The immunogenicity of Ad4-CSP was also tested in homologous and heterologous prime boost vaccinations in both Ad5 naïve and Ad5 immune backgrounds as compared to use of Ad5-CSP.

**Results:**

In Ad5 naïve animals, use of Ad4-CSP priming vaccinations followed by boosting with Ad5-CSP (Ad4-CSP/Ad5-CSP) maximally increased the numbers of CSP specific cytokine secreting cytotoxic T cells relative to repeated use of Ad5-CSP. The Ad4-CSP/Ad5-CSP regimen also induced equivalent levels of CSP specific cell killing as did homologous prime-boost vaccinations with Ad5-CSP, despite stimulating lower numbers of CSP specific cytotoxic T cells. Priming with Ad4-CSP followed by a homologous boost resulted in significantly less CSP specific humoral responses than any other vaccination regimen tested in Ad naïve animals. In Ad5 immune animals, addition of Ad4-CSP in homologous or heterologous prime boost resulted in inductions of higher CSP specific responses than animals repeatedly vaccinated with Ad5-CSP alone. However, the observed responses were well below those observed in similarly treated Ad naïve mice.

**Conclusions:**

While the Ad4-CSP/Ad5-CSP and Ad5-CSP/Ad5-CSP vaccination regimens resulted in equivalent CSP specific killing in Ad naïve animals, Ad4-CSP/Ad5-CSP achieved this result with a lower percentage of CSP specific CD8^+^ T cells and a higher number of IFNγ secreting cells, suggesting that the Ad4-CSP/Ad5-CSP vaccination regimen elicits more efficient cytotoxic T cells. In Ad5 immune animals use of Ad4-CSP improved CSP specific immune responses as compared to repeated use of Ad5-CSP, but could not achieve the levels of immunogenicity observed when the same vaccine regimens were used in Ad naïve animals. These data indicate the existence of some level of immunological cross-reactivity between these two adenovirus subgroups. Based on these results, it is suggested that future studies should undertake similarly stringent analyses of alternative Ad serotypes to establish their effectiveness as replacements for Ad5.

## Background

Despite use of prophylactic medications and vector control, malaria continues to be one of the world’s most deadly health concerns claiming the lives of almost 1 million people annually. The protozoan parasite, *Plasmodium falciparum*, accounts for about 90% of these deaths [1]. Numerous *P. falciparum* targeted vaccine studies are currently underway in efforts to eliminate this dangerous killer. The *P. falciparum* derived circumsporozoite protein (CSP) is the most studied and commonly used antigen for the purpose of developing a vaccine against malaria [2-6]. CSP is abundant on the surface of the sporozoite, and is also present in the plasma membrane and cytosol of plasmodium infected hepatocytes. CSP is a 58 kD protein composed of a C-terminus containing the thrombospondin-like type I repeat region (TSR involved in liver sinusoid attachment), a central region of [NANP] repeats, and a N-terminal site that when in contact with the liver sinusoid is cleaved exposing the TSR [7-9].

Of the several malaria vaccine vectors that target CSP, the most successful to date is a vaccine formulation that consists of a novel fusion protein between the hepatitis B surface protein (HBsAg) and CSP, and additional adjuvants. This formulation, referred to as RTS,S/AS01B, is currently in a phase 3 clinical trial [10]. This vaccine has been able to confer protection to 56% of vaccinated individuals [3,10-15]. Although promising, the results also suggest that more potent immune responses may be required to achieve higher levels of protection. For this reason other vectors and immunogenic strategies incorporating CSP are being pursued in efforts to develop a highly efficacious, malaria specific vaccine.

Recombinant adenovirus serotype 5 (rAd5) based vaccines are important in this regard as they have been confirmed to elicit potent adaptive responses against expressed transgenes [16-18]. Multiple studies have utilized rAd5s genetically engineered to express CSP in human and mouse models of malaria [6,19,20]. However, pre-existing Ad5 immunity is common in regions where malaria is endemic, and the presence of neutralizing antibodies against Ad5 has been shown to hinder Ad5 based vaccine efficacy [21-23]. It has been hypothesized that the use of alternative serotype based rAds may induce improved immunogenic responses to antigens irrespective of pre-existing Ad5 immunity, for example in HIV vaccine development [24,25]. There are at least 52 different human Adenovirus serotypes. Adenovirus serotypes are divided into subgroups A-F based primarily on anti-sera neutralization. Since Ad5 is a member of subgroup C, it is hypothesized that alternative serotypes from other subgroups would not be neutralized by Ad5 antibody and therefore, could still be utilized to infect cells and stimulate immunity to an expressed transgene. Use of alternative serotype based Ad vectors can serve other important purposes aside from stimulating immune responses in Ad5 immune patients. Heterologous prime boost regimens where the prime and boost vaccinations are derived from two different Ad serotypes based vaccines can provide greater inductions of immunity than homologous prime boosting with a single Ad serotype based vaccine [26-28].

In this context, Ad4 based vectors may be promising for use in malaria specific applications. The efficacy and safety of Ad4 vaccine platforms has been established. For instance, as the principal serotype causing Acute Respiratory Disease (ARD) in military recruits, an orally administered, live Ad4 virus was utilized for decades in vaccinations of recruits against ARD [29-32]. More recently, Ad4 based vaccines have been successfully utilized in HIV vaccine strategies in dog and chimpanzee models [24,25]. This research article analyses the ability of an Ad4-based malaria specific vaccine expressing CSP to stimulate potent immune responses when used in homologous or heterologus prime boost regimens with an Ad5 vaccine also expressing CSP, both in the context of Ad5 naïve and Ad5 immune animals.

## Methods

### Vector construction

The open reading frame (ORF) of the *P. falciparum* CSP gene, composed of a codon optimized consensus of several *P. falciparum* CSP sequences, was incorporated into an E1, E3 deleted adenovirus serotype 5 vector under the control of a cytomegalovirus (CMV) enhancer/promoter element as previously described [33]. The same CSP consensus sequence was incorporated into an E1, E3 deleted adenovirus serotype 4 vector under the expressional control of the same CMV enhancer/promoter element. Ad4 vector construction was performed as previously described with an Ad4 recombination based production system [34].

### Animal procedures

All animal procedures were approved by the Michigan State University Institutional Animal Care and Use Committee (IACUC). 6–8 weeks old male BALB/cJ mice were injected intramuscularly (IM) into the tibialis anterior of the right hind limb. Total injected volume was 20 μl. Splenocytes and plasma were collected. All procedures with rAds were performed under BSL-2, and all vector treated animals were maintained in ABSL-2 conditions. Care for mice was provided in accordance with PHS and AAALAC standards.

### ELISA

ELISA-based antibody assays were completed as previously described [16]. High-binding flat bottom 96-well plates were coated with 0.2 μg of purified CSP per well in a volume of 100μL and incubated overnight at 4°C. Plates were washed with PBS-Tween (0.05%) then treated with blocking buffer (3% bovine serum albumin) for 1 hour at room temperature. Plasma from ad naïve animals was diluted (1:100, 1:200, 1:400) in blocking buffer. Plasma from Ad immune animals was analysed without dilution. Samples were incubated for 1 hour at room temperature. Wells were washed with PBS-Tween (0.05%) and HRP antibody (Bio-Rad) was added at 1:4,000 dilution in PBS-Tween. Tetramethylbenzidine (TMB) (Sigma-Aldich) was added to each well and the reaction was stopped with 2 N sulfuric acid. Plates are read at 450 nm in a microplate spectrophotometer. Sub-isotyping titering was completed with a hybridoma sub-isotyping kit (Calbiochem, La Jolla, CA) with plasma dilutions of 1:100, 1:200 and 1:400. Statistical analyses were performed using Student *t*-test.

### Isolation of lymphocytes

Splenocytes from individual mice were prepared by physical disruption of the spleen. The spleen was passed through a sterile 40 μm nylon mesh cell strainer (Fisher Scientific, Pittsburgh, PA). Red blood cells were lysed using *ACK* lysis *buffer* (Invitrogen, Carlsbad, CA) remaining cells were resuspended in RPMI 1640 supplemented with 10% FBS and penicillin/streptomycin/fungizone [35].

### ELISPOT analysis

ELISpots were performed in accordance to manufacturer’s protocol using the Ready-set Go IFNγ mouse ELISpot kit produced by eBiosciences (San Diego, CA). Splenocytes were stimulated *ex vivo* with 4 μg/mL of the >98% pure CSP immunodominant peptide NYDNAGTNL (amino acids 43–51 of the CSP sequence) (GenScript Piscataway, NJ) [36]. Spots were counted and photographed by an automated ELISPOT reader system (Cellular Technology, Cleveland, OH). Ready-set Go IFNγ and IL-2 mouse ELISPOT kits purchased from eBioscience (San Diego, CA).

### Cell staining and flow cytometry

Splenocytes were stained with various combinations of the following antibodies: APC-Cy7-CD3, Alexa Floure700-CD8, PerCpCy5.5-CD127, PE-Cy7-CSP (NYD) tetramer, V450-CD62L, PE-Cy7-TNFα, APC-IFNγ, and Granzyme B- (4 μg/ml) (All obtained from *BD Biosciences*, San Diego, CA). Cells were incubated on ice with the appropriate antibodies for 30 minutes, washed, and sorted using an LSR II instrument and analysed using FlowJo software. For intracellular cytokines staining, cells were surface stained, fixed with 2% formaldehyde (Polysciences, Warrington, PA), permeabilized with 0.2% saponin (Sigma-Aldrich, St. Louis, MO), and stained for intracellular cytokines. Large cells and debris were excluded in the forward- and side-scatter plot, to minimize background levels of staining caused by nonspecific binding of antibodies; cells were initially stained with CD16/32 FcR III/II antibody. In addition the violet fluorescent reactive dye (ViViD, Invitrogen) was used as a viability marker to exclude dead cells from the analysis [37]. Blood was isolated by retro-orbital bleeds and PBMCs were isolated using Lympholyte-Mammal (Cedarlane, Burlington NC). Tetramer staining of PBMCs was completed using a PE conjugated MHC-I (H2d) tetramer folded with the NYDNAGTNL peptide generated at the NIH Tetramer Core Facility.

### In vivo CTL assay

BALB/cJ were injected with a priming dose of 1×10^10^ vp/mouse of either Ad5-CSP or Ad4-CSP followed by a heterologous or homologous boosting injection of 1×10^10^ vp/mouse 14 days after the initial injection. At 28 days post initial injection, syngeneic splenocytes were isolated and pulsed with either an irrelevant peptide or peptide specific to the *P. falciparum* circumsporozoite antigen (NYDNAGTNL) for 1 hour at 37°C. Irrelevant peptide pulsed cells were stained with 1 μM CFSE (CFSE^Low^) while CSP-peptides pulsed cells were stained with 10 μM CFSE (CFSE^High^). Naïve and immunized mice were injected with equivalent amount of both CFSE^Low^ and CFSE^High^ stained cells via the retro-orbital sinus. After 18 hours splenocytes were harvested and sorted on an LSRII flow cytometer. FlowJo software was used to determine percentages of CFSE stained cells. % Specific killing = 1-((% CFSE^High^ / % CFSE^Low^) _immunized_ / (% CFSE^High^ / % CFSE^Low^) _non-immunized_).

### Statistical analysis

Statistically significant differences in ELISpot assays were determined using either Two Way ANOVA with a Bonferroni post-hoc test or a One Way ANOVA with a Student-Newman-Keuls post-hoc test (p value < 0.05). For ELISA analysis, a t-test was used to assess significance between treatments. For multiparameter flow cytometry, a One Way ANOVA with a Student-Newman-Keuls post-hoc test was used. For *in vivo* CTL assay, a One Way ANOVA with a Student-Newman-Keuls post-hoc test was used. All graphs in this paper are presented as Mean ± SD with the exception of ELISA graphs, which use Mean ± SE. GraphPad Prism software was utilized for statistical analysis.

## Results

**Figure 1:**
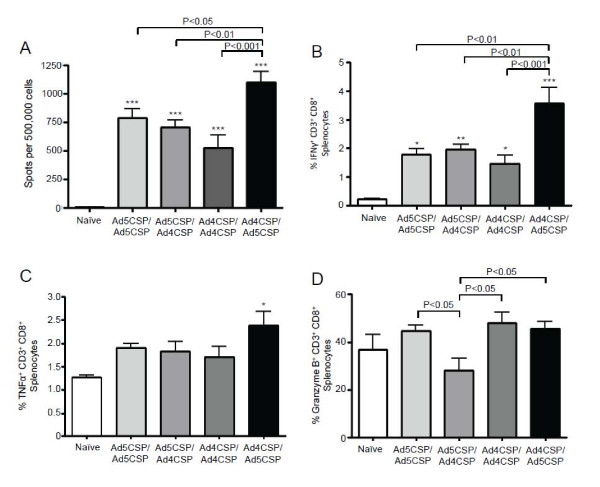
**Ad4-CSP/Ad5-CSP heterologous prime boost results in improved quality of T cell response.** A prime injection of 1 × 10^10^ vp/mouse Ad4-CSP followed by a boost of 1 × 10^10^ vp/mouse of Ad5-CSP resulted in significantly more IFNγ secretion by splenocytes measured by ELISpot (**A**) and CD3^+^ CD8^+^ T cells measured by flow cytometry (**B**). Ad4-CSP/Ad5-CSP was the only treatment to stimulate a significantly higher percentage of TNFα production as compared to unvaccinated animals (**C**). Cells were stained with CD3-APC-Cy7, CD8-Alexa flour700, TNFα-PE-Cy7, IFNγ-FITC, and Granzyme B-APC. Bars represent ± standard error. Statistical analysis was completed using One Way ANOVA with Student-Newman-Keuls post-hoc test, *, **, *** denotes significance over naïve, *P* < 0.05, *P* < 0.01, *P* < 0.001.

rAds of serotype 4 and serotype 5 were engineered to express a codon optimized form of CSP using methods previously described [33,34]. Four vaccination regimens were utilized; 1. Ad5-CSP/Ad5-CSP, 2. Ad5-CSP/Ad4-CSP, 3. Ad4-CSP/Ad4-CSP, and 4. Ad4-CSP/Ad5-CSP, where the Ad serotype used in the priming vaccination is immediately followed by the serotype of the boosting vaccination in each vaccine regimen or group. Initially, (day 0) Ad naïve BALB/cJ mice were injected with either Ad4-CSP or Ad5-CSP (1x10^10^ vp/mouse) (n = 10). 14 days later 5 mice from each treatment group received a homologous boost (same Ad-CSP serotype vaccine) of 1x10^10^ vp/mouse, the other five mice from the same group received a heterologous boosting vaccination of 1x10^10^ vp/mouse with the alternative Ad-CSP serotype vaccine. 28 days after the priming vaccinations, splenocytes were harvested from the animals and stimulated with the CSP derived peptide (NYDNAGTNL) and the number of IFNγ secreting splenocytes were quantified by ELISpot. While every vaccine treatment resulted in a significant increase in the numbers of CSP responsive INFγ secreting splenocytes when compared to non-vaccinated animals, the Ad4-CSP/Ad5-CSP heterologous prime boosting vaccine treatment group induced significantly higher numbers of IFNγ secreting splenocytes than any other treatment group (Figure 1A). Of note, previous experiments have confirmed that simple administration of Ad vaccines does not significantly increase numbers of IFNγ secreted cells, for example when splenocytes derived from Ad vaccine treated animals are stimulated with control peptides [33-35]. These results were further supported by intracellular staining with antibodies against CD3, CD8, and IFNγ, as the percentage of CSP responsive CD3^+^ CD8^+^ IFNγ^+^ cells present in splenocytes derived from mice vaccinated with the Ad4-CSP/Ad5-CSP regimen were significantly higher when compared to splenocytes from animals treated with the other vaccination strategies (Figure 1B). Intracellular staining was also performed to enumerate the frequency of TNF and Granzyme B producing CD8^+^ T cells present in the spleens of the variously vaccinated animals. Again, the Ad4-CSP/Ad5-CSP experimental vaccination regimen appeared to confer the most robust immune responses against CSP, as it was the only treatment to induce significantly higher percentages of CSP responsive CD3^+^ CD8^+^ TNF^+^ cells as compared to non-vaccinated animals (Figure 1C). Interestingly, none of the vaccination strategies induced significantly higher percentages of CSP responsive CD3^+^ CD8^+^ Granzyme B^+^ cells as compared to non-vaccinated animals; however, animals from the Ad5-CSP/Ad4-CSP vaccination group had significantly lower percentages of CD3^+^, CD8^+^, Granzyme B^+^ T cells as compared to all other treatment groups (Figure 1D).

**Figure 2:**
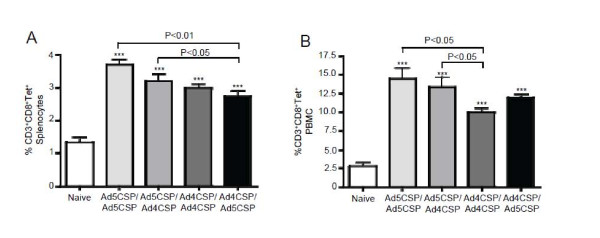
**Ad5-CSP/Ad5-CSP vaccination resulted in higher percentage of tetramer positive CD8**^**+**^**T cells than Ad4-CSP/Ad5-CSP in the spleen.** Splenocytes and PBMCs were collected two weeks after final vaccination. All vaccination regimens resulted in significantly higher percentage of CD3^+^ CD8^+^ NYDNAGTNL tetramer positive T cells in the spleen (**A**) and circulating blood (**B**) as measured by flow cytometry, cells were stained with CD8-Alexa flour700, CD3-APC-Cy7, and CSP (NYD)-Tetramer-PE. Ad5-CSP/Ad5-CSP stimulated a higher percentage of CD3^+^ CD8^+^ tet^+^ than Ad4-CSP/Ad5-CSP treated animals in the spleen (**A**) and higher percentage of CD3^+^ CD8^+^ tet^+^ than Ad4-CSP/Ad4-CSP in the circulating blood (**B**). Bars represent ± standard error. Statistical analysis was completed using One Way ANOVA with Student-Newman-Keuls post-hoc test, *, **, *** denotes significance over naïve, *P* < 0.05, P < 0.01, *P* < 0.001.

As detected by use of the NYDNAGTNL tetramer, each of the vaccination regimens induced significantly higher percentages of CD3^+^ CD8^+^ T cells in the spleen as compared with non-vaccinated control animals (*p* < 0.001) (Figure 2A). Of the four groups, the Ad4-CSP/Ad5-CSP heterologous prime boosting regimen induced the lowest percentage of CD3^+^ CD8^+^ tet^+^ T cells, a decrease that was statistically significant as compared to both the Ad5-CSP/Ad5CSP and the Ad5-CSP/Ad4-CSP treatment groups (*p* < 0.01; *p* < 0.05 respectively). When peripheral blood mononuclear cells (PBMCs) from the vaccinated mice were similarly analysed, again all groups of vaccinated mice had significantly increased numbers of CD3^+^ CD8^+^ tet^+^ T cells present as compared to non-vaccinated mice. However, the Ad4-CSP/Ad4-CSP treated animals elicited the lowest percentages of CD3^+^ CD8^+^ tet^+^ T cells of the four groups, this decrease reaching statistical significance when this group was compared to both the Ad5-CSP/Ad5-CSP and Ad5-CSP/Ad4-CSP treatment groups (*p* < 0.05 for each group) (Figure 2B).

**Figure 3:**
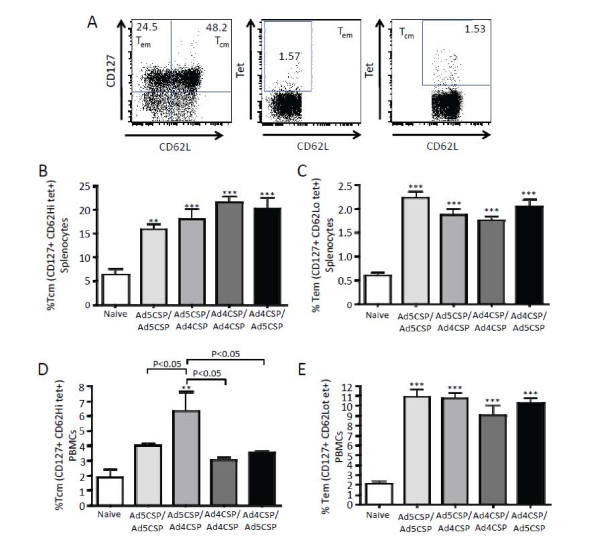
**Memory responses triggered by vaccination with homologous and heterologous prime boost regimens utilizing Ad4-CSP and Ad5-CSP in Ad naïve mice.** Splenocytes (**B**-**C**) and PBMCs (**D**-**E**) were collected two weeks after final vaccination. Cells were stained for CD62L-V450, CD127-PerCP Cy5.5, and CSP (NYD) tet-PE. CSP specific central memory T cells were determined as CD62L^+^ CD127^+^ cells that are tet^+^ and effector memory cells are CD62L^lo^ CD127^+^ cells that are tet^+^. Provided above is an example of gating (**A**). Bars represent ± standard error. Statistical analysis was completed using One Way ANOVA with Student-Newman-Keuls post-hoc test, **, *** denotes significance over naïve, *P* < 0.01, *P* < 0.001.

Ad vectors are known to elicit strong T_em_ cell responses thought to be due to more persistent antigen production. This is important in the context of a malaria vaccine as T_em_ cell responses have been shown to be beneficial in protecting against liver stage malaria [38]. The magnitude of CSP-specific central memory and effector memory CD8^+^ T cell responses was compared in each of the various prime boost regimens induced in splenocytes and PBMCs harvested 14 days after the boosting vaccinations. All prime boost regimens demonstrated much higher percentages of CSP specific T_cm_ cells and T_em_ cells than was observed in non-vaccinated animals as indicated by the percent of CD127^+^ CD62L^+^ and CD127^+^ CD62L^-^ tet^+^ T cells present in the splenocytes (Figure 3B-C). The percentage of CSP specific T_cm_ and T_em_ cells circulating in the blood was also analysed and it was found that the Ad5-CSP/Ad4-CSP vaccination group was the only group that had a significantly higher percentage of CSP specific T_cm_ cells in circulating blood when compared to non-vaccinated animals, while all vaccinated animals had higher percentages of CSP specific T_em_ cells present in this compartment as compared to non-vaccinated animals (Figure 3D-E). When memory phenotypes were analysed by gating on tetramer positive cells first, followed by gating for CD127 and CD62L, the tetramer positive cells of all groups had similar memory phenotypes as defined by comparison of the percentages of tet^+^ cells that were T_em_ and those that were T_cm_ cell.

Splenocytes from all treatments were analyzed for the presence of anti-Ad4 and/or anti-Ad5 antigen specific IFNγ secreting T cells by ELISpot. There was no significant cross stimulation between the two serotypes detected by this assay, as animals that received Ad4-CSP/Ad4-CSP treatment had significantly less Ad5 specific IFNγ secreting cells than all other vaccination regimens, and were not significantly different than naïve animals. Likewise, animals that were vaccinated with Ad5-CSP/Ad5-CSP had significantly less Ad4 specific IFNγ secreting cells than animals that received Ad4-CSP injections and were also not significantly different than naïve animals ( Additional file 1).

**Figure 4:**
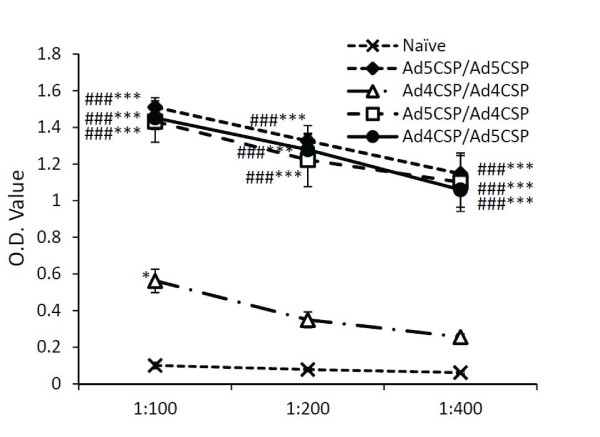
**All vaccinations stimulated significantly higher anti-CSP total IgG than unvaccinated and AD4-CSP/Ad4-CSP vaccination in Ad naïve animals.** Plasma was collected 14 days post the final vaccination. Plasma was diluted 1:100, 1:200, and 1:400 and measured for total IgG against CSP by ELISA. Bars represent ± standard error. Statistical analysis was completed using One Way ANOVA with Student-Newman-Keuls post-hoc test, *, **, *** denotes significance over naïve, *P* < 0.05, *P* < 0.01, *P* < 0.001. ### denotes significance over Ad4-CSP/Ad4CSP treatment, *P* < 0.001.

The effect of prime boost vaccinations combining Ad4-CSP and Ad5-CSP on CSP specific antibody production, as compared to homologous prime boosts using the same vectors was then determined. Plasma was collected from BALB/cJ mice injected with the four prime boost regimens 28 days post initial injection and was tested by ELISA for total anti-CSP IgG antibody levels. Mice from the Ad4-CSP/Ad4-CSP vaccination group demonstrated significantly higher plasma levels of IgG anti-CSP relative to unvaccinated animals at the 1:100 dilutions (*p* < 0.05) (Figure 4). All other vaccination regimens induced significantly higher levels of anti-CSP IgG as compared to both the non-vaccinated animals and animals receiving the Ad4-CSP/Ad4-CSP regimen (*p* < 0.001) (Figure 4). Similar trends were observed when sub-isotyping analysis was performed for anti-CSP IgG1, IgG2a, IgG2b, and IgG3 levels ( Additional file 2). IgG2a/IgG1 ratio was analysed as an indirect assessment of T_h_1 vs. T_h_2 immune responses in animals treated with the vaccine regimens; however the T_h_1/T_h_2 ratio was not significantly different with use of any of the vaccination regimens ( Additional file 3).

**Figure 5:**
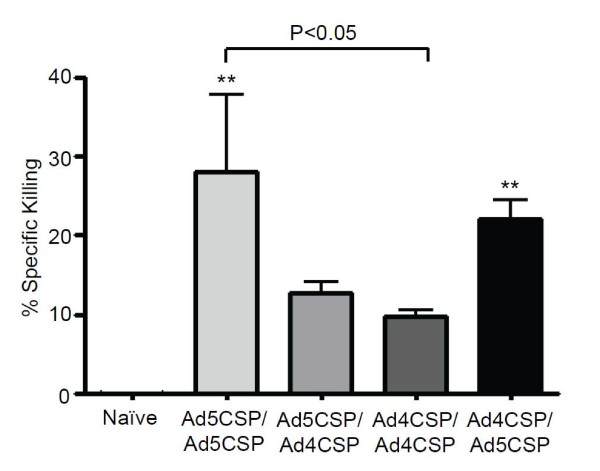
**Ad5-CSP/Ad5-CSP and Ad4-CSP/Ad5-CSP both stimulated more percent specific killing than unvaccinated animals.** 14 days post vaccination splenocytes from naïve animals were pulsed with either NYDNAGTNL and high concentration of CFSE or non-specific peptide and low concentration of CFSE. Stained splenocytes were combined in equal amounts and roughly 8 million cells were injected into vaccinated animals IV. After 20 hours splenocytes from vaccinated mice were collected and analysed by flow cytometry to assess the amount of NYDNAGTNL specific killing. % Specific killing = 1-((%CFSE^high^/%CFSE^low^)_immunized_/(%CFSE^high^/CFSE^low^)_non-immunized_. Bars represent ± standard error. Statistical analysis was completed using One Way ANOVA with Student-Newman-Keuls post-hoc test, **denotes significance over naïve, *P* < 0.01.

To assess the efficacy of Ad4 based vaccination regimens to induce functional, CSP specific cytolytic T cell responses, CSP specific cytotoxic T lymphocyte killing *in vivo* was measured. BALB/cJ mice were vaccinated with the homologous and heterologous prime boost regimes as described above. 28 days after the initial vaccination, splenocytes from naïve mice were collected and incubated with either a high concentration of CFSE (10 μM) and NYDNAGTNL peptide or a low concentration of CFSE (1 μM) and a non-specific peptide. Stained and peptide pulsed splenocytes were then mixed at equal quantities and injected intravenously into vaccinated or non-vaccinated animals. After 18 hours, CSP specific cell killing was measured in the spleens of the vaccinated animals by flow cytometry. Only animals that received the Ad5-CSP/Ad5-CSP and Ad4-CSP/Ad5-CSP vaccination regimens achieved significantly elevated levels of CSP specific cell killing as compared to non-vaccinated animals (*p* < 0.01) (Figure 5).

**Figure 6:**
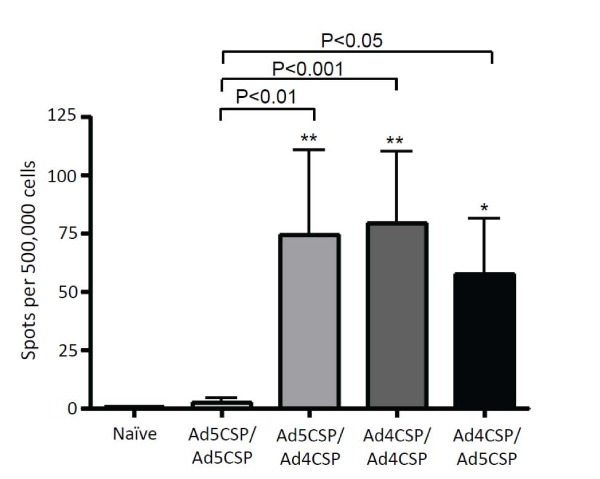
**IFNγ secretion of cells from Ad5 immune mice vaccinated with heterologous and homologous prime boost regimens utilizing Ad4-CSP and Ad5-CSP.** All vaccinations were capable of stimulating significantly more IFNγ secreting cells than unvaccinated and Ad5-CSP/Ad5-CSP vaccinated Ad5 immune animals. Splenocytes were collected 14 days after final vaccination. Splenocytes were then stimulated with CSP dominant antigen NYDNAGTNL and IFNγ secretion was measure by ELISpot. Bars represent ± standard error. Statistical analysis was completed using One Way ANOVA with Student-Newman-Keuls post-hoc test, *, ** denotes significance over naïve, *P* < 0.05, *P* < 0.01.

Given the high seroprevalence of wild type Ad5 in adults living in malaria endemic regions, the ability of these homologous and heterologous prime boost vaccine regimens to elicit potent CSP specific adaptive responses was also analysed in animals that were made Ad5 immune prior to receipt of the various vaccine regimens. BALB/cJ mice received two injections 14 days apart of 1x10^10^ vp/mouse of an Ad5 vector that does not express a transgene (Ad5-Null). It has been previously demonstrated that two immunizations with 1x10^10^ vps of rAd5-Null vector induced Ad5 neutralizing antibodies titers that were >1/200, a level that closely parallels levels of pre-existing Ad5 immunity noted in human populations [39]. 14 days after the last injection of Ad5-Null, Ad5-immune animals received 1×10^10^ vp/mouse prime injection of either Ad4-CSP or Ad5-CSP followed by either a heterologous or homologous boost 14 days after the initial priming vaccination. 28 days after the prime vaccination plasma, PBMCs, and splenocytes were collected. Splenocytes were stimulated as before with NYDNAGTNL and were analyzed for CSP specific IFNγ secreting cells by ELISpot. Ad5-CSP/Ad4-CSP, Ad4-CSP/Ad4-CSP, and Ad4-CSP/A5-CSP vaccinated Ad5 immune animals all had significantly higher numbers of NYDNAGTNL responsive IFNγ secreting cells present when compared to the Ad5-CSP/Ad5-CSP cohort or the non-vaccinated animals (Figure 6). However, as compared to Ad5 naive animals, overall induction of NYDNAGTNL responsive, IFNγ secreting splenocytes was notably diminished in Ad5 immune animals despite use of Ad4-CSP in some of the regimens (Table 1). The reductions prevented detection of significant differences between the treatments when ICS of the splenocytes for IFNγ, TNF, and Granzyme B was undertaken ( Additional file 4A-C).

**Table 1:** Decreased mean spot forming cells in Ad5 immune animals

**Vaccination**	**Ad5 naive**	**Ad5 immune**
Naive	2.71	2.71
Ad5‒CSP/Ad5‒CSP	791.25	2.80
Ad5‒CSP/Ad4‒CSP	708.00	74.50
Ad4‒CSP/Ad4‒CSP	527.20	79.60
Ad4‒CSP/Ad5‒CSP	1100	57.60

All vaccinations, inclusive of homologous Ad4-CSP prime boost, elicited fewer IFNγ secreting splenocytes in Ad5 immune animals as measured by ELISpot. The table displays the mean numbers of spot forming cells per 500,000 splenocytes from spleens of Ad5 naïve and Ad5 immune mice treated with each prime boost regimen.

**Figure 7:**
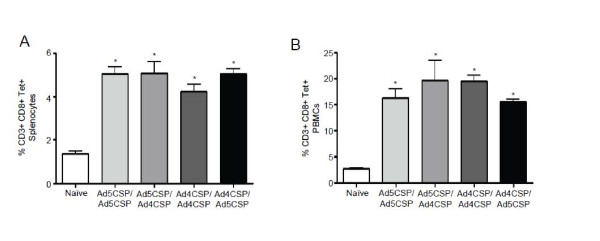
**All vaccinations in Ad5 immune animals resulted in significantly higher percentages of CD8**^**+**^**CSP tetramer positive cells than unvaccinated Ad5 immune animals.** Splenocytes and PBMCs were collected two weeks after final vaccination. All vaccination regimens resulted in significantly higher percentage of CD3^+^ CD8^+^ NYDNAGTNL tetramer positive T cells in the spleen (**A**) and circulating blood (**B**) as measured by flow cytometry, cells were stained with CD8-Alexa flour700, CD3-APC-Cy7, and CSP (NYD)-Tetramer-PE. Bars represent ± standard error. Statistical analysis was completed using One Way ANOVA with Student-Newman-Keuls post-hoc test, * denotes significance over naïve, *P* < 0.05.

**Figure 8:**
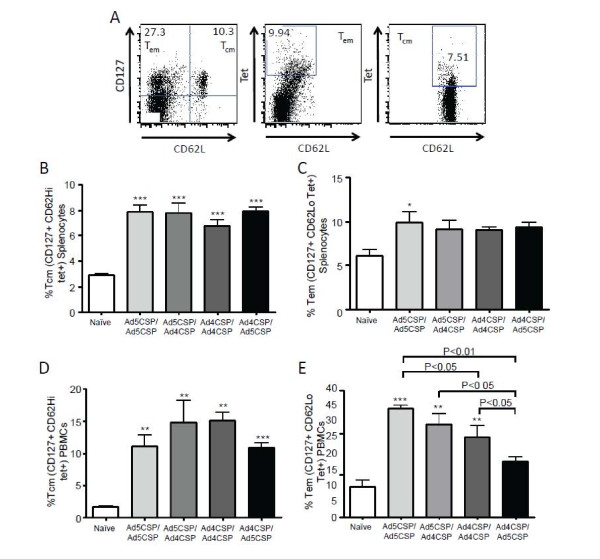
**Memory responses triggered by vaccination with homologous and heterologous prime boost regimens utilizing Ad4-CSP and Ad5-CSP in Ad5 immune animals.** Splenocytes (**B-C**) and PBMCs (**D-E**) were collected two weeks after final vaccination. Cells were stained for CD62L-V450, CD127-PerCP Cy5.5, and CSP (NYD) tet-PE. CSP specific central memory T cells were determined as CD62L^+^ CD127^+^ cells that are tet^+^ and effector memory cells are CD62L^lo^ CD127^+^ cells that are tet^+^. Provided above is an example of gating (**A**). Bars represent ± standard error. Statistical analysis was completed using One Way ANOVA with Student-Newman-Keuls post-hoc test, *, **, *** denotes significance over naïve, *P* < 0.05, P < 0.01, *P* < 0.001.

**Figure 9:**
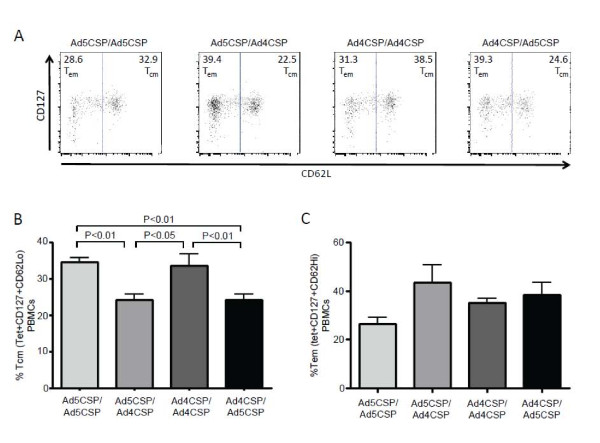
**Memory Phenotype.** Homologous prime boost regimens favor a T_cm_ cell phenotype in the peripheral blood of Ad5 immmune mice. Memory phenotype was defined as percent of CSP (NYD) tetramer positive cells that are T_em_ cells (CD62L^lo^ CD127^+^) and percentage that are T_cm_ cells (CD62L^+^ CD127^+^) as opposed to percent of T_em_ and T_cm_ cells that are Tet^+^. PBMCs were collected on day 14 post final injection and stained according to the above defined memory phenpotype. Example of gating appears above the graphs (**A**). Percentage of T_cm_ Cells was significantly higher PBMCs from homologously boosted animals in Ad5 immune mice (**B**). There was no significant difference in the percentage of T_em_ cells present between any of the groups (**C**). Bars represent ± standard error. Statistical analysis was completed using One Way ANOVA with Student-Newman-Keuls post-hoc Test.

**Figure 10:**
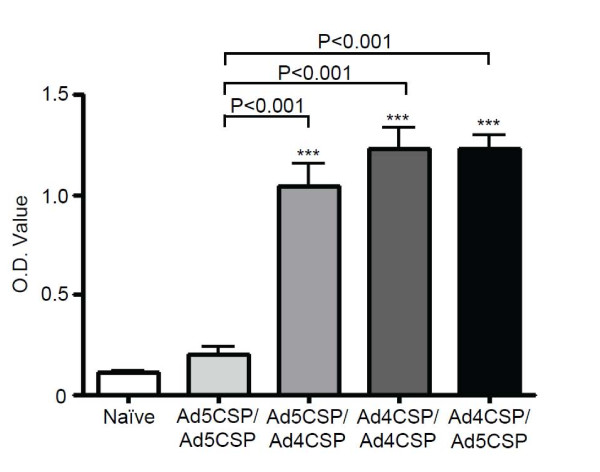
**All vaccinations stimulated significantly higher anti-CSP total IgG than unvaccinated and Ad5-CSP/Ad5-CSP vaccination in Ad5 immune animals.** Plasma was collected 14 days post the final vaccination. Plasma was measured undiluted for total IgG against CSP by ELISA. Serial dilutions were not possible as the undiluted plasma data point required the majority of the plasma collected from an animal. Bars represent ± standard error. Statistical analysis was completed using One Way ANOVA with Student-Newman-Keuls post-hoc test, *** denotes significance over naïve, *P* < 0.001.

PBMCs and splenocytes were then analysed for CD3^+^ CD8^+^ T cells that were CSP peptide tetramer binding by flow cytometry and found that all vaccinated Ad5 immune animals, including Ad5-CSP/Ad5-CSP vaccinated animals, had a significantly higher percentage of CSP specific CD3^+^ CD8^+^ tet^+^ T cells present in both the spleen and peripheral blood (Figure 7A-B). All treatments including Ad5-CSP/Ad5-CSP also had significantly higher percentages of tetramer positive T_cm_ cells when compared to the non-vaccinated animals in both the spleen and in the peripheral blood (Figure 8B, D). Only mice from the Ad5-CSP/Ad5-CSP treatment group had higher frequencies of CSP specific T_em_ cells in their spleens as compared to non vaccinated mice (Figure 8C). Ad5-CSP/Ad5-CSP, Ad5-CSP/Ad4-CSP, and Ad4-CSP/Ad4-CSP vaccination groups all stimulated significantly more T_em_ cells in the peripheral blood than non-vaccinated and Ad4-CSP/Ad5-CSP vaccinated Ad5 immune-animals (Figure 8E). T cells memory phenotypes were measured and it was found that homologous prime boost vaccinations biased the T cell responses toward T_cm_ rather than T_em_ cell phenotype memory in Ad5 immune mice (Figure 9). The *in vivo* cytolytic activity of CD8^+^ T cells in Ad5 pre-immune mice was also evaluated. No significant increase in percent specific killing was observed in any treatment groups when compared to unvaccinated Ad5 immune animals (data not shown). Undiluted plasma collected from unvaccinated animals and Ad5 immune animals from each vaccination regimen was analysed for anti-CSP total IgG by ELISA. From the undiluted plasma, it was found that Ad5-CSP/Ad4-CSP, Ad4-CSP/Ad4-CSP, and Ad4-CSP/A5-CSP vaccinated animals all had significantly more CSP specific total IgG than non-vaccinated animals (*p* < 0.001) and the Ad5 immune animals homologously vaccinated with Ad5-CSP (*p* < 0.001) (Figure 10).

## Conclusions

Ad4 has many qualities that make it a desirable choice as a vaccine platform, inclusive of an ability to induce robust early innate responses and a high rate of infectivity [34]. Ad4 also has a long history of use as a vaccine being used as an enteric live Ad4 vaccine by the military to vaccinate recruits against ARD [29-32]. For these reasons Ad4 has already been utilized as a potential HIV vaccine vector in several large animal HIV models [24,25]. This study investigated how Ad4 based vaccines might be incorporated into malaria vaccine regimens, either in isolation, or in combination with a first generation Ad5 vaccine platform.

The combination of a priming vaccination of Ad4-CSP boosted by Ad5-CSP in Ad5 naïve animals results in induction of higher levels of activated CD8^+^ T cells than any other vaccination regimen used in this study. The activated T cells induced by an Ad4-CSP priming vaccination boosted by Ad5-CSP are also capable of potent CSP specific killing to levels that are equivalent to use of Ad5-CSP homologous vaccinations, despite the fact that animals homologously vaccinated with Ad5-CSP had higher levels of CSP specific CD8^+^ T cells detectable by staining with antibodies for CD3, CD8, and tetramer specific for CSP. These data suggest that combined use of Ad4-CSP priming followed by an Ad5-CSP boosting vaccination induced more efficient cytotoxic T cell killers than those induced by homologous prime boost of Ad5-CSP. If the aim is to provide a large quantity of CSP reactive T cells, a homologous prime boost vaccination of Ad5-CSP should be utilized. However, if one wishes to elicit IFNγ and TNF secreting T cells specific for CSP, these results suggest that a priming vaccination with Ad4-CSP followed by a boosting vaccination with Ad5-CSP may be preferable.

While Ad4-CSP provided benefit when utilized as a priming vaccination prior to boosting with Ad5-CSP, Ad4 was not as capable as Ad5-CSP when attempting to stimulate potent CSP specific immune responses in homologous prime boost regimens. Additionally, boosting a prime of Ad5-CSP with Ad4-CSP induced very poor CSP specific immune responses in general. Diminished induction by Ad4 based vaccines of transgene-specific IgG has been previously observed, and the effect was suggested to be a result of the Ad4 capsid inducing high levels of IFN-β, interfering with the CMV promoter used to drive expression of the antigen encoding transgene. Interference with the CMV promoter may ultimately reduce the length of time the CSP antigen is expressed from Ad4 vaccine platforms, and may explain the decrease in efficacy when Ad4-CSP is utilized in isolation or as a boosting vaccination in current studies [34].

To obtain protection from liver stage malaria, the presence of T_em_ cells are thought to be an essential element and a significant correlate to predicting vaccine efficacy [38]. Among the vaccination regimens, the induction of CSP specific T_em_ cell and T_cm_ cells were grossly similar when Ad4 or Ad5 based CSP vaccine treatments were conducted in Ad naïve animals. However, vaccination regimens did not perform equally when *in vivo* cytotoxicity was tested, as only the animals receiving an Ad4-CSP priming vaccination boosted by Ad5-CSP, and the animals receiving the homologous Ad5-CSP prime-boost vaccination regimens resulted in detection of significantly improved levels of CSP specific cell killing, as compared to non-vaccinated animals. Since 14 days post vaccination is within the time frame when peak CD8^+^ effector T cell responses may be present, and CD8^+^ T cell contraction usually does not take place until after three weeks post-vaccination, it is likely that the observed CSP specific killing is a result of the lingering presence of CD8^+^ effector T cells, rather than induction of T_em_ cells.

Another reason to undertake these studies was to determine if the use of serologically distinct Ad4 based malaria targeted vaccines might allow for improved induction of CSP immune responses, relative to repeated use of Ad5, in Ad5 immune animals. Indeed, Ad4-CSP was capable of stimulating the induction of significantly more CSP antigen specific IFNγ secreting splenocytes, as well as higher levels of anti-CSP antibodies when incorporated into prime boost regimens in Ad5 immune mice (as compared to use of the Ad5-CSP vaccine). However, these levels were below what was observed when the Ad4-CSP vaccine was utilized in Ad5 naïve animals. Furthermore, functional analysis suggested that use of Ad4 in Ad5 immune animals also did not result in improved induction of CSP specific cytotoxic activity as compared to non-vaccinated animals. Although Ads are segregated into subgroups based primarily on anti-sera neutralization there is evidence that T cell responses can react across Ad subgroups in humans [40]. These cross reactive T cells could be responsible for the decreased immunogenicity observed in Ad5 immune animals homologously vaccinated with Ad4-CSP. Therefore, *a priori* assumptions regarding lack of likely cross neutralization by utilizing different subgroups of adenovirus must be reconsidered in light of these types of functional, in vivo data. Likely, mild cross reactivity not measured by conventional means (such as the neutralizing antibody and ELISpot based assays used in this study) is still capable of diminishing immunogenicity of two very distinct serotypes on the basis of perhaps only a few cross reactive epitopes [41].

Prior immunity to Ad5 did not appear to affect the ability of any of the Ad4-CSP or Ad5-CSP vaccination regimens to induce CSP specific CD8^+^ T cells. The percentages of CSP specific tetramer positive CD8^+^ T cells observed in Ad5 immune animals were similar to percentages observed in Ad naive animals. All vaccinations appeared to increase the percentage of CD8^+^ T cells specific to the CSP epitope NYDNAGTNL in Ad5 immune animals in spite of the observed ablation in cytokine production by these same cells. Similarly, all vaccinations except an Ad4-CSP prime boosted by Ad5-CSP resulted in high percentages of CSP specific T_em_ cells in the circulating blood. Heterologous prime boost vaccinations even appear to trend toward a T_em_ cell phenotype while homologous vaccinations biased toward a T_cm_ cell phenotype. However, none of these responses correlated with evidence of improved *in vivo* CSP specific cytotoxic T cell killing when either of these vectors were deployed into Ad5 immune animals. Ad5 cross reactivity with Ad4 appears to result in the ablation of IFNγ and TNF secreting CSP specific cytotoxic T cells induction by Ad4-CSP based vaccines, despite allowing for the induction of high percentages of CSP specific T cells.

The data shows that there exists a complex interaction between immune responses triggered by a rAd4 (subgroup E) and those triggered by rAd5 (subgroup C), each expressing the same malaria antigen, in this instance, CSP. While combined use of Ad4-CSP priming vaccinations with Ad5-CSP boosting vaccinations results in the induction of greater numbers of CSP responsive cytokine secreting, cytotoxic T cells in Ad5 naïve animals, there appears to be interference between the two seemingly distinct Ad subgroups, resulting in diminished inductions of transgene specific immune responses in Ad5 immune animals despite the use of the Ad4 platform. Future studies need to be performed to further elucidate the mechanism behind Ad4’s decreased ability to stimulate immune responses in an Ad5 immune background. Based on these results it is important that future use of alternative Ad serotypes be scrutinized under similarly stringent assay conditions to ascertain their true effectiveness in overcoming pre-existing Ad5 immunity.

## Competing interests

The authors declare that they have no competing interests.

## Authors’ contributions

NS Carried out animal injections, performed all ELISA, ELISpot and *in vivo* CTL assays, assisted with Ad5-CSP design and development, and drafted the manuscript. YA performed all other flow cytometry experiments. SG designed and developed Ad4-CSP and assisted with animal work. SS assisted with design and development of Ad4-CSP. YK designed and developed Ad5-CSP. AA conceived of the study and participated in design and coordination. All authors read and approved the final manuscript.

## Supplementary Material

Additional file 1 **Ad4-CSP/Ad4-CSP and Ad5-CSP/Ad5-CSP vaccinated animals have no significant cross stimulation of splenocytes.** Splenocytes were collected 14 days post final vaccination and were stimulated with either heat inactivated Ad4-Null or heat inactivated Ad5-Null. Animals treated with Ad5-CSP/Ad5-CSP were not significantly different from naïve animals when stimulated with heat inactivated Ad4-CSP as measured by IFNγ secretion by ELISpot. Likewise, animals treated with Ad4-CSP/Ad4-CSP were not significantly different from naïve animals when stimulated with heat inactivated Ad5-CSP as measured by IFNγ secretion by ELISpot. Bars represent ± standard error. Statistical analysis was completed using One Way ANOVA with Student-Newman-Keuls post-hoc test, *, **, *** denotes significance over naïve, *P* < 0.05, *P* < 0.01, *P* < 0.001. (PDF 99 kb) Click here for file

Additional file 2 **Sub-isotype analysis of IgG antibody from plasma of mice vaccinated with heterologous and homologous prime boost regimens utilizing Ad4-CSP and Ad5-CSP.** Plasma was collected 14 days post final vaccination. The amount of CSP specific subisotype IgG1 (A), IgG2a (B), IgG2b (C), and IgG3 (D) were analysed by ELISA. Bars represent ± standard error. Statistical analysis was completed using One Way ANOVA with Student-Newman-Keuls post-hoc test, *, **, *** denotes significance over naïve, *P* < 0.05, *P* < 0.01, *P* < 0.001. (PDF 114 kb) Click here for file

Additional file 3 **Th1 to Th2 ratio (IgG2a/IgG1) of plasma from vaccinated Ad naïve animals.** Plasma was collected 14 days post final vaccination. The amount of CSP specific IgG subisotypes was measured by ELISA. Th1 to Th2 ratio was determined by dividing O.D. values from IgG2a and IgG1. Bars represent ± standard error. Statistical analysis was completed using One Way ANOVA with Student-Newman-Keuls post-hoc test, * denotes significance over naïve, *P* < 0.05. (PDF 49 kb) Click here for file

Additional file 4 **CD8**^**+**^**T cell activation in Ad5 immune animals vaccinated with heterologous or homologous prime boost regimens utilizing Ad4-CSP and Ad5-CSP.** Splenocytes were collected from vaccinated animals 14 days post the final vaccination. Cells were stained with CD8-Alexa flour700, CD3-APC-Cy7, TNFα-PE-Cy7, IFNγ-FITC, and Granzyme B-APC and analysed by flow cytometry for INFγ secreting CD3^+^ CD8^+^ T cells (A), TNFα secreting CD3^+^ CD8^+^ T cells (B), and granzyme B^+^ CD3^+^ CD8^+^ T cells (C). Bars represent ± standard error. Statistical analysis was completed using One Way ANOVA with Student-Newman-Keuls post-hoc test, *, **, *** denotes significance over naïve, *P* < 0.05, *P* < 0.01, *P* < 0.001. (PDF 111 kb) Click here for file
