# Supplementary figures and images for: Immunogenicity when utilizing adenovirus serotype 4 and 5 vaccines expressing circumsporozoite protein in naïve and Adenovirus (Ad5) immune mice
Source: Malar J. 2012 Jun 21;11:209. doi: 10.1186/1475-2875-11-209 (PMC3472263; doi:10.1186/1475-2875-11-209)

### Stimulated with Ad4-Null

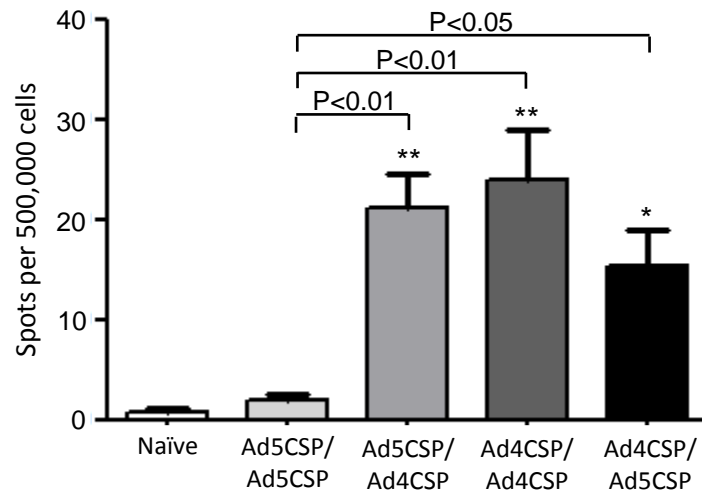

### Stimulated with Ad5-Null

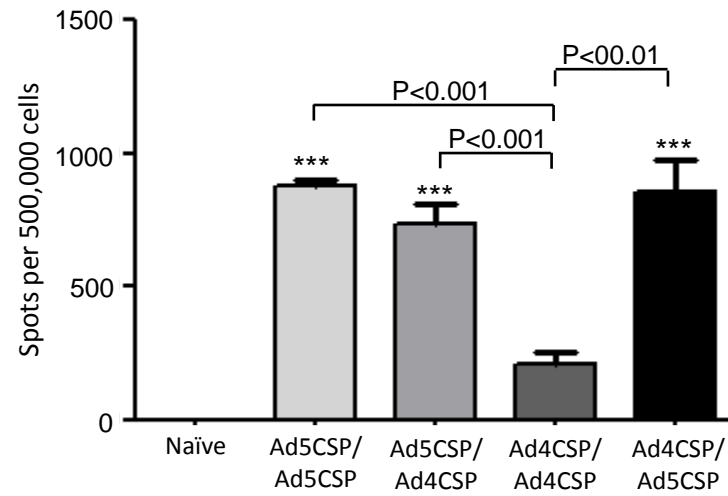

Supplement: Additional file 1 — Ad4-CSP/Ad4-CSP and Ad5-CSP/Ad5-CSP vaccinated animals have no significant cross stimulation of splenocytes. Splenocytes were collected 14 days post final vaccination and were stimulated with either heat inactivated Ad4-Null or heat inactivated Ad5-Null. Animals treated with Ad5-CSP/Ad5-CSP were not significantly different from naïve animals when stimulated with heat inactivated Ad4-CSP as measured by IFNγ secretion by ELISpot. Likewise, animals treated with Ad4-CSP/Ad4-CSP were not significantly different from naïve animals when stimulated with heat inactivated Ad5-CSP as measured by IFNγ secretion by ELISpot. Bars represent ± standard error. Statistical analysis was completed using One Way ANOVA with Student-Newman-Keuls post-hoc test, *, **, *** denotes significance over naïve, P < 0.05, P < 0.01, P < 0.001. (PDF 99 kb) [file 1475-2875-11-209-S1.pdf]

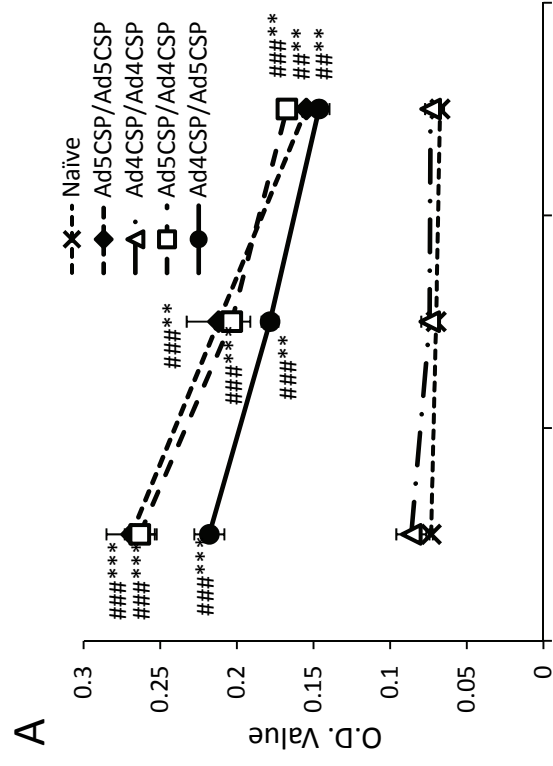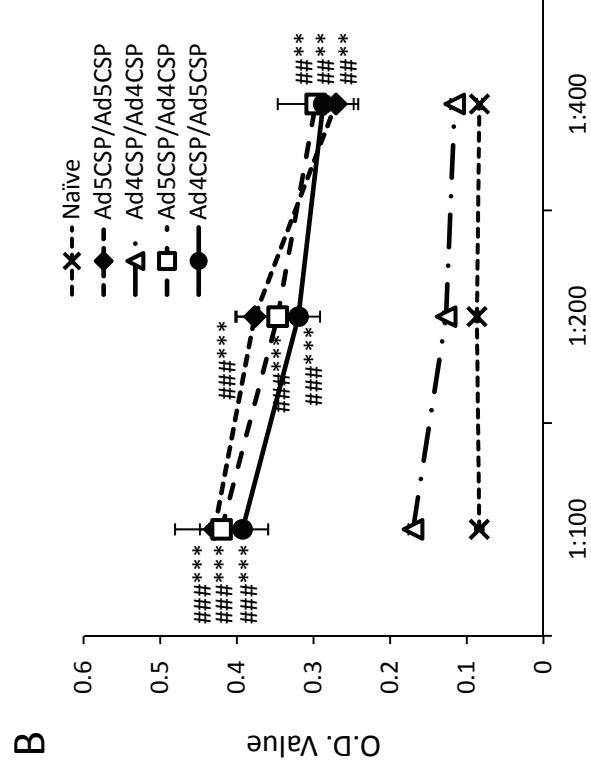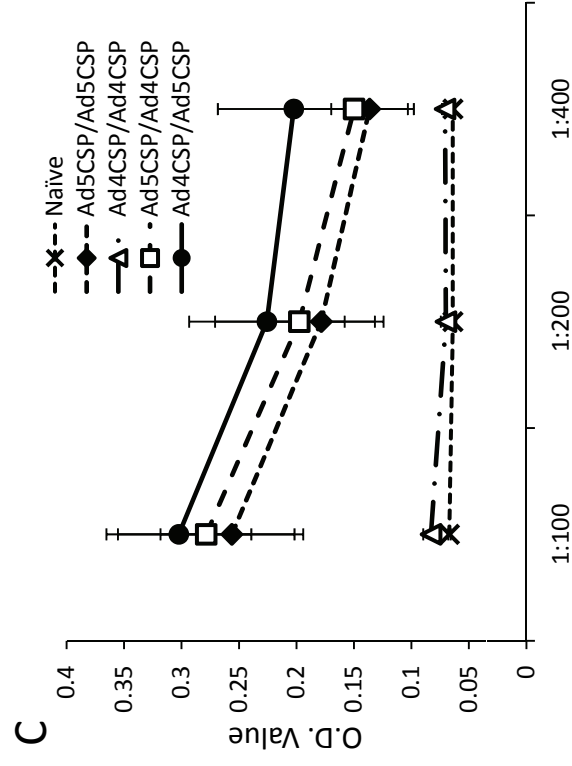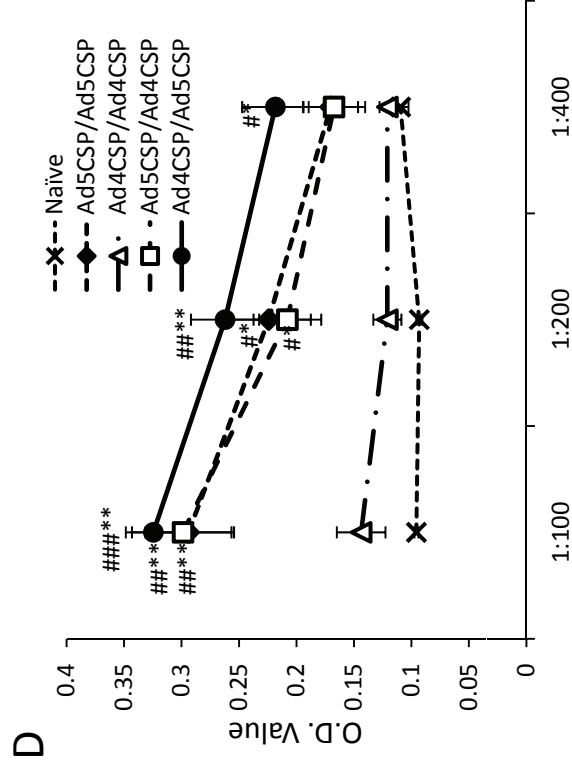

Supplement: Additional file 2 — Sub-isotype analysis of IgG antibody from plasma of mice vaccinated with heterologous and homologous prime boost regimens utilizing Ad4-CSP and Ad5-CSP. Plasma was collected 14 days post final vaccination. The amount of CSP specific subisotype IgG1 (A), IgG2a (B), IgG2b (C), and IgG3 (D) were analysed by ELISA. Bars represent ± standard error. Statistical analysis was completed using One Way ANOVA with Student-Newman-Keuls post-hoc test, *, **, *** denotes significance over naïve, P < 0.05, P < 0.01, P < 0.001. (PDF 114 kb) [file 1475-2875-11-209-S2.pdf]

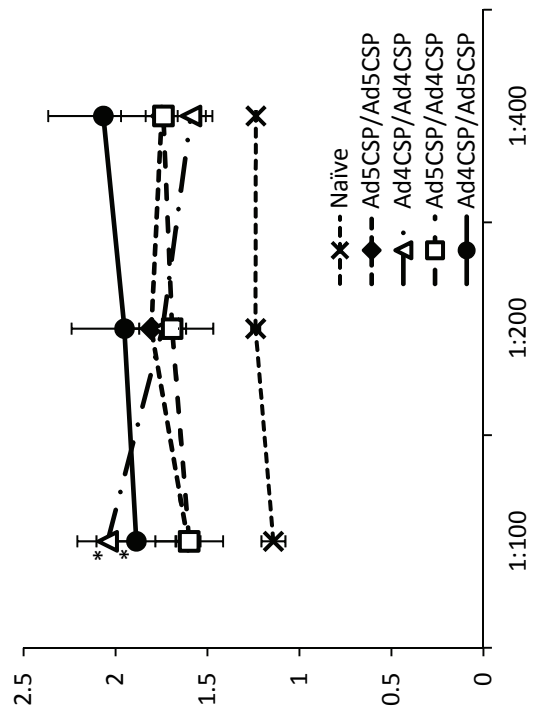

Supplement: Additional file 3 — Th1 to Th2 ratio (IgG2a/IgG1) of plasma from vaccinated Ad naïve animals. Plasma was collected 14 days post final vaccination. The amount of CSP specific IgG subisotypes was measured by ELISA. Th1 to Th2 ratio was determined by dividing O.D. values from IgG2a and IgG1. Bars represent ± standard error. Statistical analysis was completed using One Way ANOVA with Student-Newman-Keuls post-hoc test, * denotes significance over naïve, P < 0.05. (PDF 49 kb) [file 1475-2875-11-209-S3.pdf]

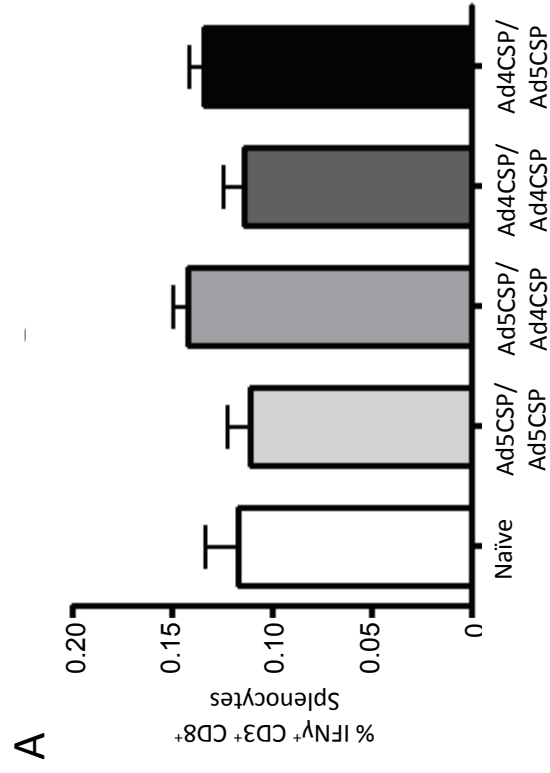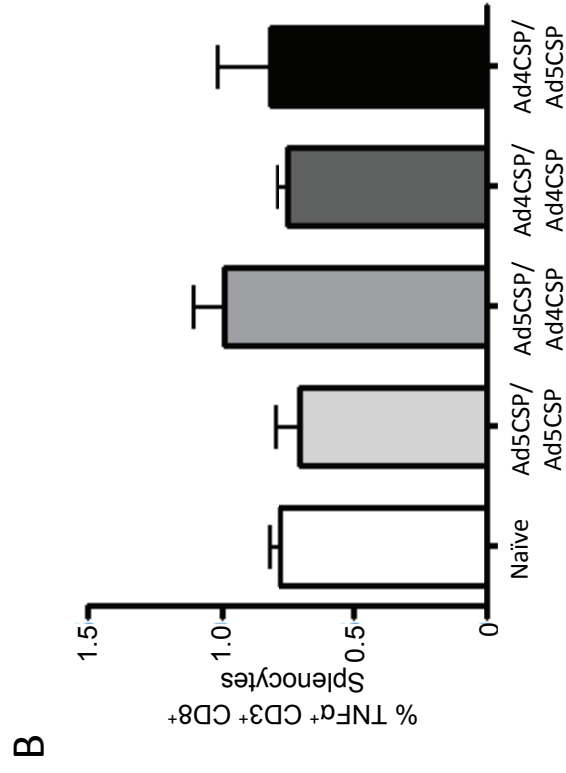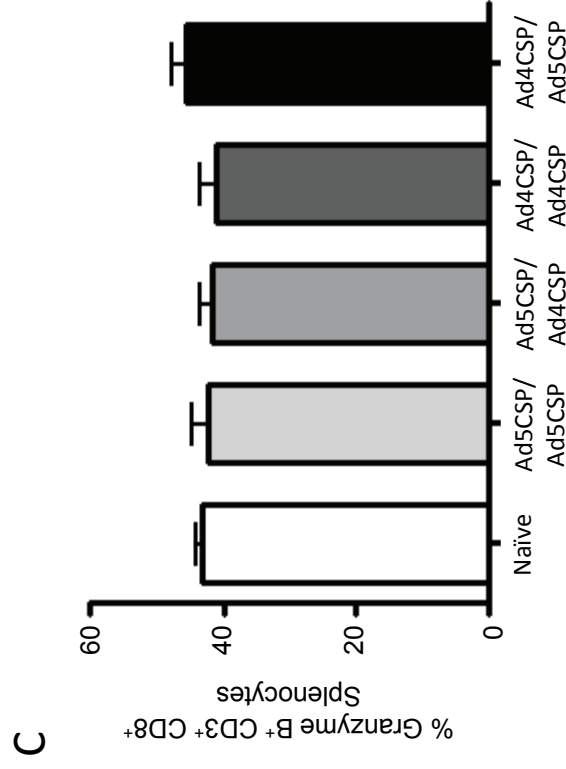

Supplement: Additional file 4 — CD8+T cell activation in Ad5 immune animals vaccinated with heterologous or homologous prime boost regimens utilizing Ad4-CSP and Ad5-CSP. Splenocytes were collected from vaccinated animals 14 days post the final vaccination. Cells were stained with CD8-Alexa flour700, CD3-APC-Cy7, TNFα-PE-Cy7, IFNγ-FITC, and Granzyme B-APC and analysed by flow cytometry for INFγ secreting CD3+ CD8+ T cells (A), TNFα secreting CD3+ CD8+ T cells (B), and granzyme B+ CD3+ CD8+ T cells (C). Bars represent ± standard error. Statistical analysis was completed using One Way ANOVA with Student-Newman-Keuls post-hoc test, *, **, *** denotes significance over naïve, P < 0.05, P < 0.01, P < 0.001. (PDF 111 kb) [file 1475-2875-11-209-S4.pdf]
